# How to Improvise a Vapor Bath

**Published:** 1893-04

**Authors:** 


### HOW TO IMPROVISE A VAPOR BATH.

Set a red hot brick on end in a can, small bath, or other suitable vessel; place the latter under a chair, on the seat of which a piece of flannel is spread. The patient, undressed, sits on this flannel, and he and the chair are well wrapped in blankets to exclude the air; his head is to be uncovered. Open the blankets a little at the bottom, and carefully pour about a pint of boiling water over the brick, and keep up the steam by occasionally repeating this. The patient remains in the bath until relieved by perspiration. To make a vapor bath in bed with hot wet bottles, fill about six oval shaped half-gallon stone bottles with boiling water; cork well, and fold each in hot wet flannel. Lay over the bed a waterproof sheet and a blanket; place the patient on these, cover him with a blanket, and distribute the hot bottles about him—one to each side, to the calf of each leg, and to the sole of each foot. Wrap up well with extra blankets, and tuck in to retain the heat. For the spirit lamp bath, place a damp towel over the seat and before the front of a cane bottom chair, under which a spirit lamp is lighted, and over the lamp a tin vessel with boiling water in it. The patient, enveloped (except the head) in four or more blankets, sits on the chair until free perspiration occurs.

